# Retraction Note: Comparative value of clinical, cytological, and histopathological features in feline mammary gland tumors; an experimental model for the study of human breast cancer

**DOI:** 10.1186/s13000-016-0574-3

**Published:** 2016-11-02

**Authors:** Radmehr Shafiee, Javad Javanbakht, Nahid Atyabi, Alimohammad Bahrami, Danial Kheradmand, Reyhaneh Safaei, Farshid Khadivar, Ehsan Hosseini

**Affiliations:** 1 Graduate, Faculty of Veterinary Medicine, Tehran University, Tehran, Iran; 2 Department of Pathology, Faculty of Veterinary Medicine, Tehran University, Tehran, Iran; 3 Paraveterinary Faculty of Ilam, University of Ilam, Ilam, Iran; 4 Graduate Student of Islamic Azad University of Mashhad, Faculty of Medicine, Mashhad, Iran

## Retraction

The Editor-in-Chief and Publisher have retracted this article [1] because the scientific integrity of the content cannot be guaranteed. An investigation by the Publisher found it to be one of a group of articles we have identified as showing evidence suggestive of attempts to subvert the peer review and publication system to inappropriately obtain or allocate authorship. This article showed evidence of plagiarism (most notably from the articles cited [2–8]) and peer review and authorship manipulation.

## References

[1] Shafiee R, Javanbakht J, Atyabi N, Bahrami A, Kheradmand D, Safaei R, Khadivar F, Hosseini E (2013). Comparative value of clinical, cytological, and histopathological features in feline mammary gland tumors; an experimental model for the study of human breast cancer. Diagn Pathol.

[2] Shafiee R, Javanbakht J, Atyabi N, Kheradmand P, Kheradmand D, Bahrami A, Daraei H, Khadivar F (2013). Diagnosis, classification and grading of canine mammary tumours as a model to study human breast cancer: an Clinico-Cytohistopathological study with environmental factors influencing public health and medicine. Cancer Cell Int.

[3] Georgieva RD, Obdeijn IM, Jager A, Hooning MJ, Tilanus-Linthorst MMA, van Deurzen CHM (2013). Breast fine-needle aspiration cytology performance in the high-risk screening population: a study of BRCA1/BRCA2 mutation carriers. Cancer Cytopathol.

[4] Millanta F, Calandrella M, Citi S, della Santa D, Poli A (2005). Overexpression of HER-2 in feline invasive mammary carcinomas: an immunohistochemical survey and evaluation of its prognostic potential. Vet Pathol.

[5] Millanta F, Lazzeri G, Mazzei M, Vannozzi I, Poli A (2002). MIB-1 labeling index in feline dysplastic and neoplastic mammary lesions and its relationship with postsurgical prognosis. Vet Pathol.

[6] Suárez-Bonnet A (2010). Martίn de las Mulas J, Millán MY, Herráez P, Rodrίguez F, Espinosa de los Monteros A. Morphological and immunohistochemical characterization of spontaneous mammary gland tumors in the guinea pig (*Cavia porcellus*). Vet Pathol.

[7] Joshi A, Maimoon S (2007). Limitations of fine needle aspiration cytology in subtyping breast malignancies – a report of three cases. J Cytology.

[8] Seixas F, Palmeira C, Pires MA, Lopes C (2007). Mammary invasive micropapillary carcinoma in cats: clinicopathologic features and nuclear DNA content. Vet Pathol.

